# Effect of maize (*zea mays l.)* and cowpea (*vigna unguiculata l.*) intercropping on agronomic performance, yield and nutritional values of maize and cowpea under supplementary irrigation

**DOI:** 10.1016/j.heliyon.2024.e39817

**Published:** 2024-10-28

**Authors:** Banso Kussie, Yilkal Tadele, Aleme Asresie

**Affiliations:** a Office of Livestock and Fishery Resource Development, Konso Zone, Karat, Ethiopia; b Department of Animal Sciences, Arba Minch University, Arba Minch, Ethiopia; c Department of Animal Sciences, Wollo University, Dessie, Ethiopia

**Keywords:** Agronomic trait, Biological competition, Chemical analysis, Economic analysis

## Abstract

A field experiment was conducted to evaluate the effect of maize (Zea mays L.) and cowpea (Vigna Unguiculata L.) intercropping on agronomic performance, yield, and nutritional values at Arba Minch University. Treatments were only maize crop (T_1_), only cowpea (T_2_), intercrop (maize and cowpea) in a 1:1 ratio (T_3_) and alternative rows of 2:2 (T4) as treatments. Treatments were arranged in a randomized complete block design (RCBD). Data on plant morphology, yield, nutritional composition, and economic return were recorded. The height of the plant (145.5 cm) of maize was significantly (P < 0.001) higher at 1:1 intercropping than the height of the sole while the height of the cowpea was higher at the sole cropping. Higher number of branches (P < 0.05) per plant (5.28) of cowpea was recorded in pure stands of cowpea than intercropping. The grain yield of maize (6.9 t/ha) and cowpea (4.2 t/ha) was significantly (P < 0.05) higher in sole cropping than in intercropping. A higher dry matter yield of 9.06 t/ha of maize was obtained in a 1:1 maize-cowpea intercropping ratio than sole maize. Significantly (P < 0.001) highest dry matter cowpea yield of 5.77 t/ha was obtained at sole cultivation than intercropping. The intercropping of maize with cowpea had an advantage of resource utilization in terms of land equivalent ratio (LER) 41.7 %–62.5 %. The marginal rate of return (MRR) of intercropping in the ratio of 1:1 was as high as 48.23 % in intercropping maize-cowpea, which was much better than sole cropping. Therefore, it could be recommended that the ratio of 1:1 maize-cowpea intercropping for land resource use and economic food and feed production in Arba Minch and similar agro-ecology zones.

## 1 Introduction

Ethiopia has the largest livestock population and has an immense potential to increase livestock production both for local use and for export [1]. However, limited feed supply and poor quality of available feeds are the main constraints for optimal livestock productivity [2]. The intercropping of forage legumes with cereal crops offers the potential to increase feed without a significant reduction in grain production [3]. Forage legumes have the ability to fix atmospheric nitrogen and contribute to the sustainability of agricultural production [4]. Cowpea (*Vigna unguiculata* L.) is an important food legume growing in the tropical and subtropical region of Africa [5]. All parts of cowpea are used as food providing protein and vitamins [6]. Cowpea is cultivated primarily in a low-land area of Ethiopia for its edible seeds, pods, and leaves. In addition, cowpea provides feed, forage, hay, and silage for livestock and green manure and cover crop, which maintain the productivity of soils [7]. Maize (*Zea mays* L) is the second most widely cultivated crop in Ethiopia and is grown under diverse agro ecologies and socioeconomic conditions [8].). It is grown main growing season known as Meher, which is based on May–September rainfall [7]. Maize is Ethiopia's leading cereal crop in terms of production with 6.2 million tons produced in 2013 by 9.3 million farmers in 2 million hectares of land [9].

Much of the feed resources available in Ethiopia are poor quality and are utilized to support the maintenance requirements of the animals with little surplus left for production [10]. An alternative strategy to improve crude protein (CP) concentrations in animal feed resources is the development of grass-legume mixtures. It has been reported that intercropping of grasses with legumes increased yield, improved growth, enhanced palatability, and nutritive quality of feeds for animals [11]. Intercropping of maize with different legumes indicated that dry matter yield and crude protein yield of forage were increased by all intercropping compositions compared with the maize monoculture [12].

Although maize-cowpea intercropping has been researched in some areas of the country, location-specific and agro ecological based evaluation of maize-cowpea intercropping for agronomic performance, forage and grain yield, nutritional values are important. Similarly, there is no information/literature on the performance of maize-cowpea intercropping in the Gamo zone in general and in the study area in particular. Therefore, this study was initiated with the objective of evaluating the effect of intercropping maize and cowpea on agronomic performance, nutritional values of maize and cowpea; and estimates the economic return of intercropping maize and cowpea.

## 2 Materials and methods

### 2.1 Description of the study area

The experiment was conducted at Arba Minch University, Kulfo Campus. The area is located about 500 km south of Addis Ababa, the capital of Ethiopia, and 275 km south west of Hawasa, the capital city of Southern Ethiopia. It is geographically located in the southern part of the east African rift valley at the absolute location between its latitude and longitude are 6^0^ 08’ North and 37° 33′ to 37° 37′ East respectively. The average annual rain falls of the area range from 920 to 1100 mm, the main rainy season is from February to May and the short rainy season from June to September Arba Minch lies at an altitude ranging between 1000 and 1350 m above sea level (m.a.s.l.) and has an average temperature of 29 °C [13].

### 2.2 Experimental design

Treatments were sole maize (T1), sole cowpea (T2), intercrops of maize-cowpea in alternate rows of 1: 1 (T3) and 2: 2 (T4). The treatments were arranged in a randomized complete block design (RCBD). The land was divided into four blocks according to the soil fertility gradient. The total number of plots was 16. The BH-546 maize variety and the Temesgen cowpea variety were used for the experiment. The growth habit of cow pea variety used was semi-erect (trailing). The plot size of the experiment was 25 m^2^ (5m∗5m). The distance between the plots and the blocks was 1.0 m and 1.5 m, respectively. The inter-row spacing of 75 cm and the plant-to-plant distance of 25 cm was used for maize. Similarly, 75 cm between rows and 20 cm between plants was used for cowpea. In the intercrops, cowpea and maize seeds were sown at 25 cm apart and inter-row spacing was 75 cm. The field experiment was carried out during from 01 January to August 01, 2023.

### 2.3 Land preparation and crop management

The land was plowed three times, making the soil fine before planting. Hoeing and hand weeding were carried out during establishment and subsequently, as deemed necessary. Supplemental flooding type irrigation was used throughout the experimental period.

### 2.4 Data collection methods

#### 2.4.1 Agronomic data

The following parameters were assessed: number of leaves per plant, plant height and biomass yield, number of cobs per plant, grain yield, and weight of 100 seeds.

##### 2.4.1.1 Plant height (cm)

To determine plant height at each harvesting time, the height of the plant was measured for randomly selected plants from middle rows using a measuring tap (cm). Ten (10) plants were randomly selected from each experimental plot at forage harvest.

##### 2.4.1.2 Number of branches per plant

Branches of 10 randomly selected plants were counted at forage harvesting for each maize and cowpea separately.

##### 2.4.1.3 Number of leaves per plant (NLPP)

The number of leaves of 10 randomly selected plants for maize and cow pea were counted separately at harvesting and converted to per plant.

##### **2.4.1.4** Leaf-to-stem ratio (LSR)

Leaf yield and forage stem yield for both maize and cow pea were weighed at harvest separately and the leaf yield to stem yield ratio was presented.

##### **2.4.1.5** Dry matter yield (DMY t/ha)

To determine the herbage yield, the total forage yield per plot was harvested from each plot at 5 cm above ground and then weighed. About 500 g of fresh sub-sample weight were randomly taken from each plot at a time of harvesting for chemical analysis. Fresh weight was taken in the field using a sensitive balance for top loading and cut into short lengths (2–5 cm) for determination of dry matter. To estimate the dry matter yield (DMY), the fresh sample was weighed and the fresh weighed sub-samples (FWss) were oven-dried (DWss) at 60 °C for 72 h and weighed. The actual dry matter was determined by drying the feed samples at 105 0c overnight. The dry matter yield per hectare was then calculated using the formula of [14]. DMY kg/ha = (10 Tot FW (DWss/HAx FWss).

Where-Total FW = total fresh weight from the plot in kg. DWss = dry weight of the sample in grams.

FWss = Fresh weight of the sample in grams, HA = harvest area square meter, 10 = is a constant for the conversation of yields in kg m^2^to ton/ha.

### 2.5 Chemical analysis

For chemical analysis, the feeds were ground to pass through 1 mm sieve size. The dry matter content (DM) was determined by oven drying the samples at 105 °C for 24 h. The ash content was determined by burning the sample at 550 °C in the furnaces. The nitrogen (N) content was determined using the Kjeldahl method according to Ref. [15] and the CP was calculated as Nx6.25. Neutral detergent fiber, acid detergent fiber (ADF), and acid detergent lignin (ADL) were determined by the methods of [16]. The in vitro digestibility of the dry matter of the offered feed and the refusals were determined according to the two-stage procedures of [17], the chemical analysis was conducted at Holetta Agricultural Research Center of animal nutrition research laboratory in Ethiopia. The metabolism energy of DOMD was estimated as ME (MJ/kg) = 0.16 ∗ in vitro digestibility of organic matter [18].

### 2.6 Economic analysis of maize-cow pea intercropping

Profitability analysis was made following the procedure of [19]. Consequently, the gross return (GR) was calculated as the income generated from selling forage crops. The net return (NR) was calculated as the difference between the income from the forages harvested and the variable costs incurred in producing forages. The following formula was used:

NR = GR-TVC; Where: NR = net return; GR = gross return; TVC = total variable cost.

Total variable costs include the costs of all inputs (labor cost, input costs like seed, fertilizer) that change due to the change in market and technology. The most important criterion in deciding whether or not to adopt a new technology is the change in net return (ΔNR). This amount is the difference between the change in gross return (ΔGR) and the change in total variable costs (ΔTVC).

*Δ*NR = *Δ*GR-*Δ*TVC The marginal rate of return (MRR) measures the increase in net income (ΔNR) associated with each additional unit of expenditure (ΔTVC) and is calculated as follows:

MRR=ΔNR/ΔTVC

For each specific case, costs, prices, and managerial assumptions were made. The following variable cost items were considered; land preparation, planting, pest, disease, and weed control, and harvesting cost. Costs were calculated per kg of dry matter produced; Seed cost is the amount of seed used as measured in kilograms multiplied by the price per kilogram. Labor costs were calculated as the wage rate multiplied by the number of man days used for planting. The costs were based on the current market prices. The selling price of the forages in birr/kg was used to estimate the net return. Selling price of forage/herbage) was estimated as follows: There is forage/herbage open market at Arba Minch town which is about 3 km from the experimental site. Weighed amount of forage material was sent to this market to get the price estimation. In the market, ten persons (feed merchants, sellers and local farmers/buyers) were separately asked to judge the price. Finally we use the mean values of price estimations forage/herbage.

### **2.7** Data analysis

The collected data were subjected to the SAS statistical software analysis of variance (ANOVA) procedure and the mean values were separated using Tukey's HSD test at P < 0.05. The model used for the analysis of biomass yield from agronomic data and chemical composition analysis was; Yij = μ + Ai + Bj + εijk*;* Where: Yijk = the response variable/observation*;μ* = the overall mean, Ai = the effect of treatment, Bj = the effect of Block, εijk = random error.

## 3 Results

### 3.1 Agronomic traits of maize under maize-cowpea intercropping

#### 3.1.1 Plant height

The mean value of plant height, leaf/cultivar number, and leaf-to-stem ratio for maize and cow pea under different intercropping is presented in Table 1. The height of the maize and cowpea plant was significantly (P < 0.001) affected by intercropping. The result revealed that the height of the maize plant was 102.75–145.5 cm and that of the cowpea was 70.4–130.8 cm (Table 1).

**Table 1:** Plant height, leaf/tiller number leaf-to-stem ratio, and forage dry matter yield of maize and cow pea.

Treatments	pH (cm)		LNPP		LSR		DMY (t/ha)	
	Maize	Cowpea	Maize	Cowpea	Maize	Cowpea	Maize	Cowpea
Intercrops 1:1	145.5^a^	70.4^c^	15.49^a^	51.8	0.46^ab^	0.288	9.06^a^	1.28^b^
Intercrops 2:2	118.9^b^	101.4^b^	14.75^b^	53.9	0.5^a^	0.4955	8.24^ab^	0.99^b^
Sole maize/cowpea	102.45^b^	130.8^a^	15.01^ab^	59.8	0.498^a^	0.4615	6.59^b^	5.77a
Mean	122.28	100.9	15.09	55.2	0.486	0.415	7.96	2.68
P-value	<0.001	<0.001	0.044	0.168	0.047	0.054	0.014	<0.001
LSD_0.05_	15.71	11.4	0.572	11.01	0.572	0.11	1.578	0.914
CV%	12.1	10.6	3.6	14.9	16.7	41.2	18.6	32

Means with same letter and columns are not significant different; (P < 0.01) = significant; (P > 0.01) = non-significant; (P < 0.05) = significant difference; (P > 0.05) = non-significant; HS = harvesting stage CS = cropping system SM = sole maize; 1:1 (intercrops of maize-cowpea in 1:1 alternative rows); 2:2 (intercrops of maize-cowpea in 2:2 alternative rows); pH = plant height; NCPP = number of cob per plant; NLPP = number of leaves per plant; LSR = leaf to stem ratio; cm = centimeter, BMY = biomass yield; DMY = dry matter yield ton ha^−1^; LSD = least significant differences and CV (%) = Coefficient of variation.

#### 3.1.2 Number of leaves/tillers per plant, leaf-to-stem ratio and dry matter yield

The mean values of the number of leaves/tillers per plant, the leaf-to-stem ratio, and the dry matter yield for maize and cowpea under different intercropping are presented in Table 1. The intercropping had a significant effect (P < 0.05) on the number of leaves per plant of maize only. The result indicated that the highest leaf number per plant of maize was obtained at the intercropping 1:1 rows (15.49) followed by sole maize (15.01) than the intercropping ratio in the intercropping of 2:2 rows (14.75).

The branching performance of cowpea was significantly affected by the cropping system (P < 0.01). The leaf to stem ratio of maize varied significantly (P > 0.05) among the different intercropping. The dry matter yield of the forage of both maize and cowpea was affected by intercropping. Higher dry matter yield of 9.06 t/ha was recorded at the 1:1 intercropping ratio followed by the 2:2 intercropping ratio (8.24 t/ha) than sole cropping (6.59 t/ha) of maize. The better yield of dry matter in intercropped maize than sole in the present study implies that intercropping modifies the microclimate for the growth and development of the maize crop.

#### 3.1.3 Number of cobs/pods per plant, 100 seed weight and grain yield

There was no significant effect of intercropping on the number of cobs per plant for maize, while the number of pods for cowpea varied for different intercropping (Table 2). A higher number of pods per plant (31.4) were obtained at an intercropping ratio of 2: 2 than 1: 1 and a sole cowpea crop. The weight of a hundred cowpea seed was found to be not significantly (P > 0.05) differed among the intercropping system. The grain yield of maize varied significantly (P < 0.05) varied among intercropping system (Table 2). The grain yield of cowpea under maize cowpea intercropping was significantly (P < 0.001) varied with intercropping treatments.

**Table 2:** Number of cob/pod per plant, 100 seed weight and grain yield of maize and cowpea.

Treatments	Grain yield t/ha		100 seed Wt.		NCPP/NPPP	
	Maize	cowpea	maize	cowpea	maize	cowpea
Sole Maize/sole cowpea	6.9^a^	4.2^a^	36.68^a^	18.3	1.463	26.2b
Maize-cow pea mix 1	5.95^ab^	0.96^b^	35.78^a^	17.6	1.6	21.79c
Maize-cow pea mix 2	5.65^b^	0.9^b^	35.45^a^	17.5	1.538	31.4a
Mean	6.17	2.02	35.97	17.8	1.53	26.46
P-value	0.038	<0.0001	0.574	0.1685	0.403	<0.0001
CV%	14.8	18	6.58	5.07	12.9	10.3

Note∗; NCPP: maize cob number per plant, NPPP: cowpea number of pods per plant.

#### 3.1.4 Nutritional composition, dry matter, in vitro digestibility and metabolic energy values of maize and cowpea

Dry matter (DM), ash, crude protein (CP), acid detergent fiber (ADF), acid detergent lignin (ADL), neutral detergent fiber (NDF), in vitro digestibility of dry matter (IvDMD), organic matter digestibility (DOMD) and metabolizable energy (ME) significantly (P < 0.01) affected by the intercropping system (Table 3).

**Table 3:** Nutritional composition, dry matter digestibility in vitro and metabolic energy values of maize and cowpea.

Parameters	Cowpea			Maize			P- value	CV%
	Sole cowpea	Maize-cowpea 1	Maize -Cowpea 2	Sole maize	Maize-cowpea 1	Maize-Cowpea 2		
DM%	93.07^a^	91.18^b^	91.22^b^	93.11^a^	93.56^a^	93.59^a^	<.0.01	0.6
ASH%	14.3^a^	9.71^bc^	8.45^cd^	5.205^ef^	5.15^ef^	6.53^de^	<.0.01	8.7
CP%	22.43^a^	24.08^a^	23.23^a^	5.02^c^	6.28^c^	5.75^c^	<.0.01	6.2
NDF%	36.30^de^	38.09^de^	39.51^cd^	72.48^a^	71.61^a^	70.59^a^	<0.001	4.0
ADF%	22.58^ef^	26.22^de^	26.23^de^	42.59^a^	39.04^ab^	40.29^ab^	<0.001	3.4
ADL%	4.32^ab^	3.65^ab^	4.57^ab^	5.505^ab^	7.435^a^	7.31^a^	0.0001	21.2
DOMD	60.06^ef^	61.29^def^	60.26^ef^	63.46^bcde^	61.27^def^	64.73^abcd^	<0.001	1.5
IVDMD	55.24^cde^	56.29^cde^	56.26^de^	54.93^de^	52.60^e^	56.27^cde^	<0.001	1.7
ME (MJ/Kg DM	9.00^cde^	9.005^cde^	8.84^de^	8.785^de^	8.415^e^	9.005^cde^	<0.001	1.7

*Note∗; Mean values in the columns without common superscripts are different at (P<0.05),* DM = Dry matter, CP=Crude protein, NDF= Neutral detergent fiber, ADF= Acid detergent fiber, ADL = Acid detergent lignin, IVDMD = Invitro dry matter digestibility, DOMD = organic matter digestibility in the dry matter, ME = Metabolizable energy, MC 1:1 mixed sample at 1:1 ratio intercropping, MC 2:2 mixed sample at 2:2 ratio intercropping, intercrops of maize - cow pea in 1:1 and 2:2 alternative rows,; CV % = Coefficient variation.

#### 3.1.5 Biological competition of intercropping

The values of land equivalent ratio (LER), relative crowding coefficient (K), aggression index (A) and competitive ratio (CR) were calculated to evaluate the efficiency of intercropping maize with cowpea forage and are presented in Table 4. In maize-cowpea intercropping, maize was treated as base crop with 1:1 and 2:2 row arrangements. In additive series of 1:1 intercropping, cowpea adds plant population per unit area and benefits are achieved as dry matter yield and grain yield of maize and cowpea.

**Table 4:** Land equivalent ratio, aggressiveness, competitive ratio, and relative crowding coefficient of maize cowpea intercropping.

Cropping system	LER	AM	AC	CRM	CRC	KM	KC
1:1	1.565	−0.0021	0.002	6.59	0.152	−3.782	0.260
2:2	1.417	−0.00162	0.00162	7.56	0.132	−4.976	0.198

LER: land equivalent ratio, AM: maize aggression, AC: cowpea aggression, CRM: maize competitive ratio, CRC: cowpea competitive ratio, KM/C: relative crowding coefficient of maize/cowpea.

#### 3.1.6 Partial budget analysis

Total fixed costs including fencing, field management from land preparation planting and weeding, irrigation supplement, and harvesting were not included in the budget analysis. Variable costs which were considered for budget calculation were labor cost, seed cost, and fertilizer costs. The grain yield and biomass yield values were used for the calculation of the revenue in Ethiopian birr (ETB). The partial budget analysis presented in Table 5, showed that the highest net revenue recorded in the maize cowpea intercropping ratio of 1: 1 was 232947.7 ETB, which is 33.13 % better than the single cowpea cropping and 12.39 % better than the single maize production. The marginal rate of return (MRR) was observed to be higher than 48.23 in maize cowpea intercropping in the MC 1: 1 ratio, which was better than sole cropping.

**Table 5:** Partial budget analysis of maize cowpea intercropping.

Descriptions	Intercropping pattern			
	Sole maize	Sole cowpea	Maize-cow pea 1	Maize-cowpea 2
Variable Costs				
Labor cost	4000	1000	2500	2500
Seed cost maize (25 kg/ha)	1000	0	500	500
Seed cost cowpea (25 kg/ha)	0	800	400	400
NPS(12.6528 ETB/kg)	1265.28	1265.28	1265.28	1265.28
Urea (10.3526 ETB/kg)	1035.26	0	0	0
Total Variable Costs (TVC)	7300.54	3065.28	4665.28	4665.28
Grain yield (t/ha)	6.9	4.2	6.91	6.55
**Biomass Yield(t/ha)**	**22.9**	**27.06**	**34.21**	**31.15**
**Revenue GY (23000 ETB/t)**	**158700**	**96600**	**158930**	**150650**
**Revenue BMY(2300 ETB/t)**	**52670**	**62238**	**78683**	**71645**
Total Revenue	211370	158838	237613	222295
Net Revenue (NR = TR-TVC)	204069.5	155772.72	232947.7	217629.7
ΔNR	48296.74	−48296.74	77175	−15318
ΔTVC	4235.26	−4235.26	1600	0
MRR	11.40349	11.40348881	48.23438	0

## 4 Discussion

### 4.1 Agronomic traits of maize under maize cowpea intercropping

#### 4.1.1 Plant height

Plant height is an important parameter contributing to yield in forage crops. This may be due to the fact that the taller a plant, the higher the amount of light energy absorbed and the higher the rate of photosynthesis, and consequently the amount of assimilate produced by the leaves [20]. The differences in plant height among the different intercropping patterns could be attributed to intercropping ratios that facilitated maize growth for light competition. The current result (102.75–145.5 cm plant height of maize) is also comparable to 132–149 cm for maize intercropped with pigeon pea [21]. On the other hand [22], reported plant height of 220.5 cm for maize intercropped with cowpea and lablab. The height of the maize plant may vary due to the variety, the agro ecology of the planting and other applied field agronomic managements applied [23]. The result of the plant height for cowpea was comparable with 45.5–254.8 cm reported in the lowlands of southern Ethiopia [24].

#### 4.1.2 Number of leaves/tillers per plant, leaf-to-stem ratio and dry matter yield

The result indicates that intercropping induces the possibility of emerging additional branches, leaf formation, elongation, and development. Similarly, a 5 to 20 number of branches per cowpea plant of cowpea were reported [24]. The branching performance of cowpea is an important consideration during the utilization of crops' resources for better forage yield and ground cover to reduce soil erosion and moisture retention [25]. Significantly (P < 0.05) higher number of branches per plant in cowpea alone than the 1:1 and 2:2 intercropping ratio could imply suppression of maize over cowpea in growth and development. This was also previously reported, as the number of legume branches was negatively affected by intercropping with maize and a higher value of the branch number per cowpea plant was recorded at sole cropping [26].

The leaf-to-stem ratio is an important factor that is helpful in determining the digestibility and palatability of any feed crop. Leaf-to-stem ratio of 0.32–0.36 for maize was reported under maize-cowpea intercropping was reported [27]. Consistent with the current study [28], reported of leaf to stem ratio for cowpea ranging from 0.49 to 0.56. The leaf-to-stem ratio (LSR) is one of the criteria for evaluating pasture grass quality because the higher proportion of leaves compared to stem indicates a better nutritive value [29].

The results of the dry matter forage yield in the current study agrees with another scholars [30] who reported intercropping induces an increase in yield in part due to the improvement of the microclimate in the field. Cowpea dry matter yield was significantly affected by intercropping (P < 0.001). The dry matter yield was significantly higher in sole cropping than in intercropping of cowpea with maize. This report is in agreement with other reports that under maize cowpea intercropping there was a higher value of dry matter (3.31 t/ha) yield of cowpea reported solely than intercropping of crops previously in Ethiopia [22]. Compared to this study, other scholars reported that intercropping maize with cowpea reduced cowpea dry matter yields of cowpea from 0.58 to 0.24 t/ha [28]. The present result of 5.77 t/ha dry matter yield of cowpea in sole cowpea was comparable to 6.32 t/ha in the Baressa watershed, Ethiopia [31], better than previous reports of 0.53 t/ha, 2.66 t/ha [26], 0.58 t/ha [28], under maize cowpea intercropping.

#### 4.1.3 Number of cob/pod per plant and 100 seed weight

The number of pods per plant ranged from 21.79 to 31.4. The cob per plant value of 1.6 is higher than the previous report of 1.08 for the similar intercropping system [32]. The highest number of pods per plant for 2:2 ratios intercropping may be due to the climbing nature of the crop made to increase the plant height and pod number rather than sole. Indeed, this result disagrees with other previous reports of [33] whose findings recorded a reduction in cowpea number of pods per plant under intercropping system compared to sole cropping.

The weight value of the hundred seed of 18.3 g in the sole recorded cowpea cultivation was not statistically varied from the cowpea intercropped with maize in the 1:1 (17.6 g) and 2: 2 (17.5 g) intercropping ratio. This result is highly consistent with the study conducted for the performance of maize intercropped with cowpea in Botswana [34] which reported no significant variation in the weight of 100 seeds of sole cowpea (15.75 g) with intercropped cowpea (16.00 g). The mean values of 100 seed weights of maize were not significantly (P > 0.05) varied between the different intercropping. The higher value (36.68 g) of 100 seed weight for maize sole cropping was close to 36.34 g reported by Ref. [35].The highest grain yield (6.90 t/ha) was obtained in sole maize compared to intercropping patterns. The highest grain yield obtained in the sole cropping of maize may be due to spatial competition for light resource as reported by another scholar [36] that the highest maize grain yield of sole maize in the intercropping system could be attributed to high plant density and less competition. The highest grain yield of cowpea (4.2 t/ha) was recorded in cowpea alone than in intercropping (0.96 t/ha at 1: 1 and 0.9 t/ha at 2: 2 intercropping). Similarly, 0.178.2 t/ha for sole cowpea and 0.048.39 t/ha for maize-cowpea intercrops reported [37].

#### 4.1.4 Nutritional composition, dry matter, in vitro digestibility and metabolic energy values of maize and cowpea

Some variables: ADL, IvDMD, DOMD and ME significantly varied due to harvesting stage effect. The result showed also quality parameters significantly responded to the interaction of harvesting stage and intercropping system except CP and ADL. Forage quality was better in milking stage than doughty stage for maize cowpea intercropping. Intercropping system was significantly improved the quality of forage in the present experiment. Sole cowpea and cowpea in both 1:1 and 2:2 intercropping ratio had higher and statistically at par mean values of CP% (23.23–25.61) with lower NDF% (30.25–72.48), ADF% (19.78–42.59) and ADL% (2.37–7.44).

IvDMD% (58.2–68.12) and DOMD% (52.6–62.69) values were significantly lower for sole cowpea and cowpea in 1:1 and 2:2 intercropping ratios. Composite analysis for forage quality of intercrops in the ratio of maize mixed with cowpea (MC) at 1:1 (14.19 %) and 2:2 (14.85 %) were shown significantly (p < 0.001) higher mean value than sole maize. The noted low values of CP (4.83 %) for sole maize followed by maize crop residue in 1:1 (6.21 %) and 2:2 (5.44 %) intercropping ratio revealed the low quality of cereal crop residues for forage supplement. Higher CP concentration with lower fiber and higher in vitro dry matter and organic matter digestibility was observed in milking stage than doughty stage of harvesting in the present study. From the current result we could notify that intercropping of maize with cowpea harvested at milking stage improved the quality of composite forage by 67.7 % in terms of CP than sole maize cropping.These results concur with the findings for legume cereal intercropping forage quality assessment by other researchers [38]. In addition, according to the quality rating of [39], premium quality forage contains 29 % or less ADF. The results from our experiment demonstrate that for cow pea sole and intercrops integrate cowpea, according to these criteria, would be considered to produce very high quality forage. In fact, since all intercropping ratios had CP concentration in excess of 24 % and IVDMD in excess of 60 %, the forage could be considered of high quality.

#### 4.1.5 Biological competition of intercropping

The main advantage of intercropping is the more efficient utilization of the available resources and the increased productivity compared to each sole crop of the mixture [40]. The land advantage of the intercropping of maize cowpea in the current study was in agreement with the previous study, which ranged from 7 to 157 % when studying the role of intercropping of maize and cowpea in yield and soil chemical properties [41]. This could show that intercropping realizes the efficient utilization of land resources, and it was previously reported in similar manner for mixed cropping of grain peas and false flax [42]. Other scholars reported that actual production exceeds expected productivity in maize cowpea intercropping [40]. The aggression index (A) value for maize has shown negative values and positive values for cowpea. On the other hand positive aggressively values for maize and negative values for cowpea [40]. The competitive ratio (CR) of the intercropping of maize with cowpea declared that maize was found to be more competitive than cowpea in the intercropping system in the ratios of 1: 1 and 2: 2 of sowing. Competition was greater in the 2: 2 planting ratio (7.56) of maize over cowpea. Similar competition was previously reported for the intercropping of maize forage legume intercropping previously [43]. Negative relative crowding coefficient values for maize were obtained in all intercropped mixtures, which was found to be similar with the result of other scholars [44]. Thus, this result also justifies that intercropping maize and cowpea could provide greater yield stability of yield than sole cropping of either crop [45]. The result of the partial budget analysis was in agreement with the study of maize pigeon pea intercropping in southern Ethiopia [45].

## 5 Conclusions

Maize-cowpea intercropping in the present study revealed improvements in crop growth, biological yield (grain yield and dry matter yield), and biological competition and nutritional quality of feed. The height of the maize and cow pea plants varied with different forms of intercropping. The dry matter yield of maize and cowpea showed variation between the experimental treatments with a high yield of maize 9.81 t/ha in a 1:1 combination of the cropping system, while 6.41 t/ha of cowpea in pure stands. The intercropping of maize cowpea in terms of dry matter yield had a land advantage over the sole cropping of individual crops with LER range of 41.7 %–62.5 % and the highest land advantage was observed in a 1:1 ratio of intercropping. The intercropping of maize with cowpea improved the quality of the composite forage by 60.73 % in terms of CP compared to the single maize cropping. In terms of economic benefit, the intercropping ratio of 1:1 with 235447.72 ETB is 33.13 % better than the sole cowpea production and 12.39 % better than the sole maize production.

## CRediT authorship contribution statement

**Banso Kussie:** Writing – review & editing, Investigation, Formal analysis, Data curation, Conceptualization. **Yilkal Tadele:** Visualization, Validation, Supervision, Software, Investigation, Formal analysis, Data curation, Conceptualization. **Aleme Asresie:** Writing – original draft, Software, Formal analysis.

## Ethical statements

Not applicable for this research as it does not involve animal or human subjects for data collection.

## Data availability statement

The data that support the findings of this study are available from the corresponding author upon reasonable request.

## Additional information

No additional information is available for this paper.

## Funding

This research did not receive any specific grant from funding agencies in the public, commercial, or not-for-profit sectors.

## Declaration of competing interest

The authors declare that they have no known competing financial interests or personal relationships that could have appeared to influence the work reported in this paper.
